# Violence and sociodemographic related factors among a sample of Egyptian women during the COVID-19 pandemic

**DOI:** 10.1186/s41935-021-00243-5

**Published:** 2021-10-18

**Authors:** Asmaa Mohammad Moawad, Eman D. El Desouky, Marwa Rashad Salem, Ahmed Sallam Elhawary, Sara M. Hussein, Fatma Mohamed Hassan

**Affiliations:** 1 Department of Forensic Medicine and Clinical Toxicology, Faculty of Medicine, Cairo University, Kasr Alainy Street, Cairo, 11562 Egypt; 2 Department of Epidemiology and Biostatistics, Faculty of Medicine, National Cancer Institute, Cairo University, Kasr Alainy Street, Cairo, 11562 Egypt; 3 Department of Public Health and Community Medicine, Faculty of Medicine, Cairo University, Kasr Alainy Street, Cairo, 11562 Egypt; 4 Qena Faculty of Medicine, South Valley University, Qena, Egypt

**Keywords:** SARS-CoV-2, Domestic violence, Women, Survey, Egypt

## Abstract

**Background:**

Violence against women is a worldwide problem that affects different social and economic classes, and this violence has almost increased with pandemics as the COVID-19 pandemic. The present survey aimed to assess the prevalence of violence against women in Egypt during the COVID-19 pandemic and to identify the relationship between sociodemographic factors and violence exposure. A total of 509 women were recruited using a self-completion e-form questionnaire.

**Results:**

The prevalence of violence experienced by women was (43.8%); the most common type was the emotional representing (96.0%) of exposed women, while sexual violence was the least common (13.5%). Violence exposure was affected significantly by residence governorates, husbands’ working status, reduced husbands’ working hours, and history of violence exposure.

**Conclusions:**

Violence against women in Egypt was increased during the COVID-19 pandemic, which raises the need for a strong and urgent anti-violence program to control this problem.

## Background

Violence against women is widely recognized as a severe human rights violation, and a critical public health problem with significant implications on physical, mental, sexual, and reproductive health (Semahegn and Mengistie 2015). Worldwide, about 35% of women are estimated to have experienced either physical and/or sexual intimate partner violence or sexual abuse by a non-partner at a point during their lives (excluding sexual harassment) (World Health Organization 2013).

As the global COVID-19 pandemic proceeds, countries are receiving committed measures to control the virus spread. Empowering individuals to adopt social distancing, commanding closure of schools and business, and restricting traveling all are undertaken measures to diminish the spread of infection (Campbell 2020). Tragically these isolation measures, in another way, resulted in the loss of the desired safety. Numerous victims of family violence (violence against women and children, and pet abuse) may presently be confronting the worst possible situation being caught up in the home with a violent offender (World Health Organization 2021).

Emergencies, crimes, and pandemics have been associated with increased interpersonal violence involving violence against women and children (Peterman et al. 2020). For example, outbreaks of Ebola virus in West Africa, Zika virus, and cholera led to local situations where domestic violence got to be more prevalent (International Rescue Committee 2019; Chandan et al. 2020).

In the present COVID-19 pandemic, there are reports from different countries suggesting the rise in violence against women (Wanqing 2020; Lattouf 2020; Bellizzi et al. 2020). The police station in China’s Jianli County (Central Hubei Province) announced that the number of intimate partner violence cases in February 2020 was tripled compared to February 2019 (Wanqing 2020). In Australia, a survey enrolling 400 frontline workers showed a 40% rise in “pleas for help” and an increase in case complexity by 70% (Lattouf 2020). In Italy, the national network of shelters for women exposed to gender-based violence (D.i.Re) reported that from 2 March to 5 April 2020, 80 shelters were contacted by 2867 which reflects a drastic rise (74.5%) on the usual monthly records of 2018 (Bellizzi et al. 2020).

There is no specific data in Egypt on whether there has been a rise in domestic violence during COVID-19 pandemic, so this study aimed to assess the prevalence of violence against women during the COVID-19 pandemic and to identify the relationship between sociodemographic factors and violence exposure.

## Methods

### Study design and population

The study was conducted through an online survey distributed through Facebook and WhatsApp applications, the most commonly used social media in Egypt. To obtain a high response rate, the researchers selected groups with large network access. Requests for permission to disseminate the survey were sent to the administrators of these groups. The researchers then posted the survey link with a statement that included its purpose and inviting the members to take part. The study spanned over 27 days from (4th May 2020) to (31st May 2020), 6 weeks after the lockdown.

### Sampling technique and sample size

The researchers used a convenience sampling technique whereby they searched for groups with a large female network on Facebook and WhatsApp. Information about the study was disseminated to these groups with a link to the study page. This link was made accessible on some Facebook and WhatsApp groups for 27 days. A total of 509 women (18 years old and older) completed the questionnaire over this period.

The survey included Egyptian women aged 18 years and older and excluded younger females and Egyptian women who do not live in Egypt during the pandemic.

### Data collection tool

A pre-tested e-form questionnaire in the Arabic language was used for data collection, which included two sections as follows:

#### Section I

Sociodemographic data: age, residence, marital status, educational level, working status, and husband working status (before and during COVID-19 pandemic).

#### Section II

Exposure to violence, types (physical, emotional, and sexual), response, and the offenders reported by the exposed women during the COVID-19 pandemic. The questions formatted in close-ended and multiple-choice options. Questions used in this section and the definitions of types and different forms of violence were adopted from the available literature Egypt demographic and health survey 2014 (Ministry of Health and Population, El-Zanaty and Associates, and ICF International 2015).

English was the primary language of the included items; two experts translated them to Arabic followed by other independent experts back to English. We examined the face and content validity after obtaining public health experts’ viewpoints. The preliminary data collection form was piloted on 30 participants to evaluate the comprehension and clarity of questions and the time required for answering the questionnaire, and there was no omission of phrases or words.

### Statistical analysis

Statistical analysis was done using Statistical Package of Social Science Software program, version 25 (IBM SPSS Statistics for Windows, Version 25.0. Armonk, NY: IBM Corp.). Numbers and percentages were used for qualitative variables. Comparison between groups was performed using the chi-square test for qualitative variables. *P* value of less than 0.05 was considered statistically significant. Logistic regression was done to assess independent factors that affect violence exposure during the COVID-19 pandemic; covariates entered into the model were all significant variables in bivariate analysis.

### Ethical considerations

All data collection procedures were handled confidentially in compliance with Helsinki biomedical ethics guidelines. The study page included the questionnaire, the study purpose, and informed consent, in addition to the following question: are you willing to participate in the survey? (If no submit form). Once the participants provided their consent, they accessed the survey. The research announcement clearly stated that the survey was examining women exposure to violence regarding its types and contributing factors during the COVID-19 pandemic. We informed the participants that their participation was voluntary and anonymous.

## Results

### Participants’ characteristics

The most common age group of participating women was 30–39 (41.8 %), more than two-thirds of them were living in urban areas (73.9%) and (52.1%) from Greater Cairo. The majority of the participants (63.1%) were married, more than half of them and husbands attained university education (52.3% and 57.9% respectively). The working participants before COVID-19 pandemic were representing 61.2% of our sample that reduced to 42.6% during the pandemic and applying the restrictions during the study time, while 97.8% of husbands were working before the COVID-19 pandemic that also reduced to 83.5% during the pandemic (Table 1).

**Table 1:** Sociodemographic characteristics of the participating women (*n* = 509)

	***n*** (%)
**Age (years)**	
18–24	97 (19.1)
25–29	128 (25.1)
30–39	213 (41.8)
≥ 40	71 (13.9)
**Residence governorates**	
Greater Cairo	265 (52.1)
Outside Greater Cairo	244 (47.9)
**Residence area**	
Urban	376 (73.9)
Rural	133 (26.1)
**Education level**	
Basic education	12 (2.4)
High school	32 (6.3)
University education	266 (52.3)
Postgraduate	199 (39.1)
**Marital status**	
Single	166 (32.6)
Married	321 (63.1)
Divorced	17 (3.3)
Widow	5 (1.0)
**Husband education (*n*** **= 321)**	
Basic education	12 (3.7)
High school	25 (7.8)
University education	186 (57.9)
Postgraduate	98 (30.5)
**Number of children (*n*** **= 343)**	
0	28 (8.2)
1–2	209 (60.9)
3–4	100 (29.2)
≥ 5	6 (1.7)
**Participants’ work and COVID-19**	
Before	313(61.5)
During	217(42.6)
**Husbands work and COVID-19 (*n*** **= 321)**	
Before	314(97.8)
During	268(83.5)

*Before*: was working before COVID-19 pandemic, *During*: still working in COVID-19 pandemic

### Exposure to violence and different types of violence

**Fig. 1:**
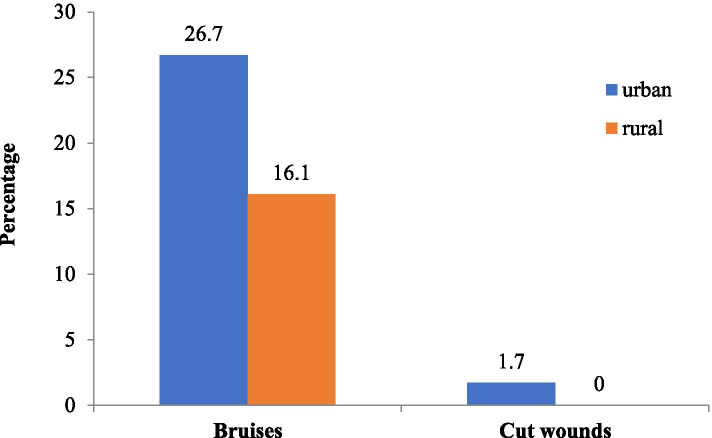
A bar chart showing the distribution of bruises and cut wounds by the residence area of women exposed to violence

Our results showed that 194 (38.1%) and 223(43.8%) of the participating women reported exposure to violence before and during the COVID-19 pandemic, respectively. The most common type of violence reported by exposed respondents during the pandemic was emotional representing (96.0%) while sexual violence represented the least common type (13.5%). Among the 30 women who experienced sexual violence, 26 were married. The most common injuries reported by the participants exposed to physical violence were bruising and cut wounds representing (24.2%) (Table 2). Figure 1 shows the distribution of bruises and cut wounds according to the residence area of exposed women, where it was more common among urban than rural areas.

**Table 2:** Violence exposure and different experienced forms of violence

Violence exposure—types	***n*** (%)
**Ever exposed to violence before COVID-19 pandemic**	194 (38.1)
**Exposed to violence during COVID-19 pandemic**	223 (43.8)
**Type of violence (*n*** **= 223)** ^**a**^	
Emotional violence	214 (96.0)
Physical violence	91 (40.8)
Sexual violence	30 (13.5)
**Emotional violence (*n*** **= 214)** ^**a**^	
Insulted her or made her feel bad about herself	163 (76.2)
Humiliated her in front of others	113 (52.8)
Threatened to harm her or harm someone she cares about	67 (31.3)
Any form of physical or sexual violence	42 (19.6)
Other forms of emotional violence	122 (57.0)
**Physical violence (*n*** **= 91)** ^**a**^	
Shook or pushed her	79 (86.8)
Twisted her arm or pulled her hair	51 (56.0)
Punched her with a fist	46 (50.5)
Slapped her	44 (48.4)
Kicked, dragged, or beat her	32 (35.2)
Threatened or attacked her with a knife or other weapon	21 (23.1)
Tried to strangle or intentionally burn her	15 (16.5)
Other forms of physical violence	35 (38.5)
**Injuries resulted from physical violence (*n*** **= 91)**	
Bruises	21 (23.1)
Cut wounds	1 (1.1)
Eye injuries	4 (4.4)
Burns	1 (1.1)
**Sexual violence (*n*** **= 30)** ^**a**^	
Forced her to have sexual relation when she did not want	27 (90.0)
Physically forced her to do any other sexual acts that she did not want	21 (70.0)
Threatened her to perform sexual acts she did not want	13 (43.3)
Other forms of sexual violence	18 (60)
**Offenders (*n*** **= 223)** ^**a**^	
Husband—ex husband	98 (43.9)
Male family member not husband	56 (25.1)
Female family member	44 (19.7)
Preferred not to answer	90 (40.4)
Non-family	32 (14.3)
**Response (*n*** **= 223)**	
Nothing	120 (53.8)
Told someone but not for help	52 (23.3)
Faced situation herself and took an action	51 (22.9)

^a^Participants reported more than one answer

**Fig. 2:**
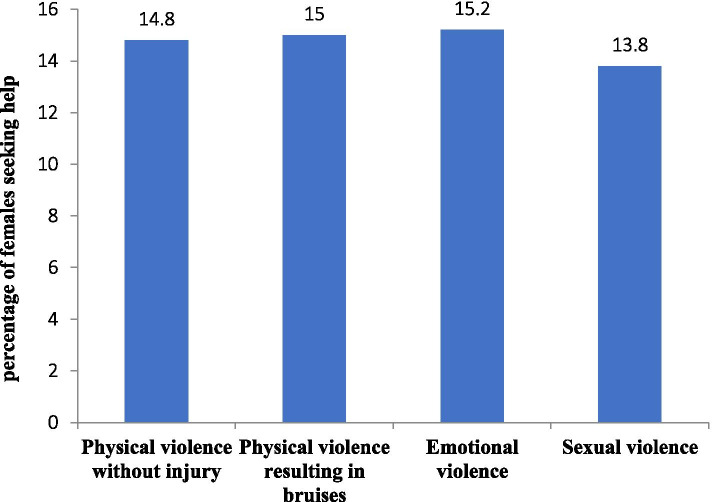
A bar chart showing the distribution of women who sought help by the type of the exposed violence

The most common offender was the husband (43.9%), whether current or ex-husband. Violence experienced by a male family member excluding husbands represented (25.1%), and by a female family member represented (19.7%). Regarding women’s response to the experienced violence, only (22.9%) faced it and took an action either in the form of divorce, asking for help, or quitting work where the abuser was found. Nearly half of them (53.8%) did not take any action **(**Table 2). Figure 2 shows the distribution of women who took an action or sought help by the type of exposed violence, with sexual violence being the least common for seeking help.

### Factors affecting violence exposure

On assessing the effects of sociodemographic characteristics on the exposure rate of violence during the COVID-19 pandemic, the residence governorates, husbands’ work, the change in husband working hours and exposure to violence before COVID-19 pandemic showed significant effects on the exposure during the pandemic (Table 3).

**Table 3:** Factors affecting violence exposure among participating women during the COVID-19 pandemic

Factors	Total	Violence (***n*** = 223)	
	***n***	(%)	***P*** value
**Age**			
18–24	97	48(49.5)	0.050
25–29	128	66(51.6)	
30–39	213	82(38.5)	
≥ 40	71	27(38.0)	
**Residence area**			
Urban	376	162(43.1)	0.579
Rural	133	61(45.9)	
**Residence governorates**			
Greater Cairo	265	100(37.7)	0.004*
Outside Greater Cairo	244	123(50.4)	
**Education level**			
Basic education	12	5(41.7)	0.396
High school	32	17(53.1)	
University education	266	122(45.9)	
Postgraduate	199	79(39.7)	
**Marital status**			
Single	166	81(48.8)	0.327
Married	321	131(40.8)	
Divorced	17	9(52.9)	
Widow	5	2(40.0)	
**Working status before COVID-19 pandemic**			
Working	313	133(42.5)	0.448
Not working	196	90(45.9)	
**Children number**			
0	28	14(50.0)	0.377
1–2	209	91(43.5)	
3–4	100	35(35.0)	
≥ 5	6	2(33.3)	
**Husband education**			
Basic education	12	5(41.7)	0.141
High school	25	14(56.0)	
University education	186	80(43.0)	
Postgraduate	98	32(32.7)	
**Participants working status during COVID-19 pandemic**			
Working	217	85(39.2)	0.069
Not Working	292	138(47.3)	
**Husband work during COVID-19 pandemic**			
Working	268	99(36.9)	0.002*
Not working	53	32(60.4)	
**Husband working hours change**			
Reduced working hours	207	96(46.4)	0.005*
Remain constant	99	29(29.3)	
Increased hours	8	1(12.5)	
**Violence before COVID-19 pandemic**			
No	315	29(7.2)	< 0.001*
Yes	194	194(100)	

*P < 0.05 is statistically significant

Odds ratio using multivariate logistic regression revealed that husband working and history of violence exposure are crucial risk factors for domestic violence during the COVID-19 pandemic. The analysis showed that women who have a history of violence exposure were 115.3 times more liable to experience violence. Moreover, women whose husbands lost their work during the pandemic and having non-working husbands were 3.9 times more likely to experience violence (Table 4).

**Table 4:** Multivariate analysis for factors that precipitate violence

	***B***	S.E.	***P*** value	OR	95% CI	
					Lower	Upper
**Violence exposure before COVID-19 pandemic**	4.747	0.417	< 0.001	115.3	50.9	261.2
**Husband work during COVID-19 pandemic**	1.35	0.524	0.01	3.9	1.4	10.8
**Constant**	− 4.098	0.732	< 0.001	0.017		

*B* regression coefficients, *SE* standard error of the coefficient, *OR* odds ratio, *95% CI for OR* 95% confidence interval for the odds ratio. *P* value < 0.05 is considered significant

## Discussion

Violent behaviors in the form of physical, sexual, psychological, and economic abuse may occur within families leading to both intimate partner violence and child abuse. These violent behaviors are more likely to increase during pandemics like COVID-19 pandemic (Peterman et al. 2020; van Gelder et al. 2020). Usually, women and girls are the first victims for violence especially domestic violence **(**Moazen et al. 2019).

This survey aimed to assess the prevalence of violence against women during COVID-19 pandemic and to identify the relationship between sociodemographic factors and violence exposure to figure out the most important preventive measures for violence reduction during the pandemic.

In this study, 38.1% and 43.8% of the responded women reported exposure to violence before and during the COVID-19 pandemic, respectively. This is consistent with reports from worldwide that revealed increasing rates of domestic violence with COVID-19 spread. Its rate has increased in the UK and USA, tripled in China, jumped to 30% increase in France, 40–50 % in Brazil, risen in Italy and Spain where home becomes often a place to experience fear and abuse (Taub 2020; Chandan et al. 2020; Campbell 2020; Marques et al. 2020).

The increased violence during the pandemic could be attributed to unemployment, reduced income, limited resources, aggressor’s increased stress, and limited social support. Victims are faced to quarantine themselves at home with potentially abusive family members. Also, women became overloaded with work in house and care for her children, elderly, and sick family members which increases rate to conflict with the aggressor (Marques et al. 2020; United Nations Population Found 2020). The “Opportunity to Abuse” is an emerging theory that may figure out the increased rates of family violence during natural disasters as the COVID-19 pandemic. It proposes that if persons capable of and willing to abuse others share the same place with those who are vulnerable to abuse as an adult, senior, kid, or animal, abuse is more likely to occur over time. With the increased chances for abuse, its likelihood of perpetration increases specifically with more vulnerable victims and low probabilities for perpetrators charging. Moreover, this theory allows abuse in any home regardless of the socioeconomic level, with increased abuse risk factors like social or cultural norms that permit abuse, drug/alcohol abuse or access, and history of abuse perpetration or victimization (Campbell 2021).

In this study, the most common type of violence experienced during the pandemic was the emotional, while the sexual represented the least common. The same finding was observed by Scott 2015, Bott et al. 2012, and Fahmy and Abd El-Rahman 2008. Also, previous studies in Egypt showed physical violence was the most common while the sexual was the least common experienced type but these studies included only married women (Ministry of Health and Population, El-Zanaty and Associates, and ICF International 2015). The reason for the decreased reporting of sexual violence may be due to the stigmatization related to this issue in certain cultures (Fahmy and Abd El-Rahman 2008).

Our results revealed that most of the women exposed to sexual violence were married. Marital rape is a global problem affecting a large number of women. It is considered legal in many countries, like South Africa and the Middle East, where most conservative societies consider it a wife’s duty. At the same time, it is criminalized in all of the USA and many Western countries that consider this act a criminal assault; this reflects the wide variation in legal and cultural concepts regarding marital rape (Yllö and Torres 2016).

Regarding injuries resulting from physical violence, bruising, and cut wounds were the most common; this was consistent with previous Egyptian studies (Ministry of Health and Population, El-Zanaty and Associates, and ICF International 2015; Badawy et al. 2014; Yaya et al. 2019). It is worthily mentioning that the highest incidence of violence fatal outcomes in 2017 was in Asia with a total of 20,000 of all women killed worldwide by intimate partners or family members, followed by Africa with a rate of 3.1 per 100,000 female population (UN Office on Drugs and Crime 2018)*.* In Egypt, Magdy and Zaki 2021 reported that in the second quarter of 2020, about 62 crimes of violence were committed against women and girls and that domestic violence crimes constituted 40.3% of the total monitored and documented crimes.

As regards the women’s reactions against violence, about half of participants did not take any response towards violent incidents. These findings agreed with many studies revealing non-reporting of women survivors of violence to police, helplines, or other service providers (Ministry of Health and Population, El-Zanaty and Associates, and ICF International 2015; Fahmy and Abd El-Rahman 2008; Tetikcok et al. 2016; Scott 2015)*.* This could be explained by fear of revenge, further violence from the offender, and feeling of embarrassment or shame (Birdsey and Snowball 2013).

Regarding the perpetrator of violence, the most common offender was the husband (current or ex), and about one-fourth of incidents committed by a male family member and 19.7% by a female offender. According to World Health Organization 2014, most violence against women is intimate partner violence. Also, Huecker et al. 2021 reported that domestic violence against women is usually perpetrated by men. On the other hand, McKeown 2014 found that female offenders of domestic violence incidents were comparable with men and may also exceed that of men (McKeown 2014).

Regarding the effect of sociodemographic factors on violence exposure, our results revealed that the residence governorates had a significant effect on exposure to violence being more common outside than inside Greater Cairo. On the other hand, being from a rural or urban area did not affect violence exposure, where it was of nearly equal frequency among urban and rural victims. A similar finding was observed by Magdy and Zaki 2021 who reported an almost equal frequency of gender-based violence cases between urban (49%) and rural cases (48%) in Egypt. They also reported a higher rate of violent crimes in Cairo, Souhag, then Qena and Qalyubia.

Exposure to violence was also significantly affected by husbands’ work, the change in husband working hours during COVID-19 pandemic, and history of violence exposure.

Multivariate logistic regression revealed that previous exposure to violence was at least 3 times more significant than job status of the husband with odds ratio 115.3 versus 3.9. The World Health Organization 2021 reported that intimate partner violence is specifically associated with a history of violence exposure. Several studies highlighted previous violence exposure as a risk factor for exposure to violence. Alkan and Tekmanlı 2021 reported that women who had suffered physical, economic, or verbal abuse had an increased likelihood of being exposed to sexual violence. According to Fisher 2004, childhood victimization increased women's chances of being victimized in high school and put women at risk for different types of dating violence. Also, Doumas et al. 1994 and Cappell and Heiner 1990 found that women who grew up in violent families were somewhat more likely to become victims of spouse abuse.

## Limitations of the study

One of the limitations was the study sample may not be representative of the female population and all standards in Egypt as the survey needed women with an online access to Facebook and WhatsApp application as COVID-19 pandemic with resulting lockdowns, social distancing policies limited the ability to interview women for survey purposes. Also, victims only, not offenders or witnesses of violence were included. Furthermore, response bias on female violence would over-represent severely affected and victimized women as participation in this survey depends on self-reported data.

## Conclusions

The prevalence of violence against women in Egypt was increased during the COVID-19 pandemic with the majority of the emotional type. The risk factors for women exposure to violence were the residence governorates, non-working status and reduced work hours for husbands, and history of violence exposure. The study recommends increasing the awareness for Professionals of Family violence victim-serving about the high possibility of increasing rates and reports of victimization both during and long after the COVID-19 pandemic and make the appropriate referral together with adequate surveillance to capture the burden of domestic violence during this pandemic. Also, the local available services information (e.g., hotline numbers, telehealth, rape support centers, and counseling) must be clear to the public through different sources, like social and mainstream media together with health facilities. The study also recommends improving women skills regarding conflict dealing and personal communications and increasing women empowerment economically and socially. Moreover, it is essential to arrange education groups and public awareness campaigns to combat harmful gender social beliefs**,** attitudes, and practices that give men the upper hand and justify violence against women.

## Data Availability

The datasets used and/or analyzed during the current study are available from the corresponding author on reasonable request.

## Abbreviations

COVID-19: Coronavirus disease-19
D.i.Re: Italian National Network of Women's Shelters
WHO: World Health Organization
SPSS: Statistical Package for Social Science program
UK: United Kingdom
